# Mineralocorticoid Receptor Antagonism Prevents Type 2 Familial Partial Lipodystrophy Brown Adipocyte Dysfunction

**DOI:** 10.3390/cells12222586

**Published:** 2023-11-07

**Authors:** Elisa Schena, Elisabetta Mattioli, Chiara Peres, Laura Zanotti, Paolo Morselli, Patricia Iozzo, Maria Angela Guzzardi, Chiara Bernardini, Monica Forni, Salvatore Nesci, Massimiliano Caprio, Carolina Cecchetti, Uberto Pagotto, Elena Gabusi, Luca Cattini, Gina Lisignoli, William Blalock, Alessandra Gambineri, Giovanna Lattanzi

**Affiliations:** 1 Unit of Bologna, CNR—National Research Council of Italy, Institute of Molecular Genetics “Luigi Luca Cavalli-Sforza”, 40136 Bologna, Italy; elisa.schena@cnr.it (E.S.); elisabetta.mattioli@cnr.it (E.M.); chiara.peres@cnr.it (C.P.); william.blalock@cnr.it (W.B.); 2 IRCCS Istituto Ortopedico Rizzoli, 40136 Bologna, Italy; 3 Unit of Gynecology and Obstetrics, Division of Endocrinology and Diabetes Prevention and Care, IRCCS Azienda Ospedaliero-Universitaria di Bologna, 40138 Bologna, Italy; laurazanotti12@gmail.com (L.Z.); carolina.cecchetti@unibo.it (C.C.); uberto.pagotto@unibo.it (U.P.); alessandra.gambineri@aosp.bo.it (A.G.); 4 Department of Medical and Surgical Sciences (DIMEC), Alma Mater Studiorum University of Bologna, 40126 Bologna, Italy; monica.forni@unibo.it; 5 Plastic Surgery Unit, Department of Specialised, Experimental and Diagnostic Medicine, Alma Mater Studiorum University of Bologna, S. Orsola-Malpighi Hospital, 40126 Bologna, Italy; paolo.morselli@unibo.it; 6 CNR—National Research Council of Italy, Institute of Clinical Physiology, 56124 Pisa, Italy; patricia.iozzo@cnr.it (P.I.); mariaangela.guzzardi@cnr.it (M.A.G.); 7 Department of Veterinary Medical Sciences, University of Bologna, 40064 Ozzano Emilia, Italy; chiara.bernardini5@unibo.it (C.B.); salvatore.nesci@unibo.it (S.N.); 8 Laboratory of Cardiovascular Endocrinology, IRCCS San Raffaele, 00163 Rome, Italy; massimiliano.caprio@sanraffaele.it; 9 Department of Human Sciences and Promotion of the Quality of Life, San Raffaele Roma Open University, 00166 Rome, Italy; 10 SC Laboratorio di Immunoreumatologia e Rigenerazione Tissutale, IRCCS Istituto Ortopedico Rizzoli, 40136 Bologna, Italy; elena.gabusi@ior.it (E.G.); luca.cattini@ior.it (L.C.); gina.lisignoli@ior.it (G.L.)

**Keywords:** type 2 familial partial lipodystrophy (FPLD2), mineralocorticoid receptor (MR), spironolactone, lamin A/C, prelamin A, adipose tissue

## Abstract

Type-2 Familial Partial Lipodystrophy (FPLD2), a rare lipodystrophy caused by *LMNA* mutations, is characterized by a loss of subcutaneous fat from the trunk and limbs and excess accumulation of adipose tissue in the neck and face. Several studies have reported that the mineralocorticoid receptor (MR) plays an essential role in adipose tissue differentiation and functionality. We previously showed that brown preadipocytes isolated from a FPLD2 patient’s neck aberrantly differentiate towards the white lineage. As this condition may be related to MR activation, we suspected altered MR dynamics in FPLD2. Despite cytoplasmic MR localization in control brown adipocytes, retention of MR was observed in FPLD2 brown adipocyte nuclei. Moreover, overexpression of wild-type or mutated prelamin A caused GFP-MR recruitment to the nuclear envelope in HEK293 cells, while drug-induced prelamin A co-localized with endogenous MR in human preadipocytes. Based on in silico analysis and in situ protein ligation assays, we could suggest an interaction between prelamin A and MR, which appears to be inhibited by mineralocorticoid receptor antagonism. Importantly, the MR antagonist spironolactone redirected FPLD2 preadipocyte differentiation towards the brown lineage, avoiding the formation of enlarged and dysmorphic lipid droplets. Finally, beneficial effects on brown adipose tissue activity were observed in an FPLD2 patient undergoing spironolactone treatment. These findings identify MR as a new lamin A interactor and a new player in lamin A-linked lipodystrophies.

## 1. Introduction

Familial partial lipodystrophy of the Dunnigan type (also called type 2 familial partial lipodystrophy, FPLD2, OMIM # 151660) is a rare genetic disease due to heterozygous mutations in the *LMNA* gene encoding lamin A/C [1,2,3]. A hot spot for FPLD2 is present at codon 482 of the *LMNA* gene (p.R482W/Q or L variant), although mutations can be found even in the protein N-terminal domain [3,4]. FPLD2 symptoms appear around puberty and consist of the loss of subcutaneous adipose tissue from the trunk and limbs and excess fat accumulation in the neck and face [5,6]. In addition, FPLD2 patients develop severe insulin resistance, hypertriglyceridemia, and metabolic complications such as diabetes mellitus, fatty liver disease, polycystic ovary syndrome, and early cardiovascular complications, in some cases associated with the accumulation of visceral adipose tissue [7,8]. Available therapeutic approaches are mostly aimed at mitigating the symptoms of the disease and improving some of the metabolic complications, but they do not prevent the worsening of the adipose tissue dystrophy [8,9].

FPLD2 is associated with impaired maturation of mutated prelamin A, which affects pre-adipocyte differentiation in part due to altered transcriptional regulation and autophagic signaling [1,10,11,12,13]. Data collected so far suggest that precursors of adipose tissue play a fundamental role in the pathogenesis of FPLD2 [1,14,15,16,17]. Under non-pathological conditions, in humans, precursors of subcutaneous adipocytes tend to differentiate mainly towards the white lineage, thus forming white adipose tissue (WAT), a fat deposit that stores energy and is involved in hormonal and inflammatory activity [18]. Brown adipose tissue (BAT) is found in the neck, supraclavicular and perirenal regions, and areas surrounding the major blood vessels, where it is associated with WAT [19,20]. Brown adipocytes are small, polygonal cells with ~50% of the volume occupied by lipids partitioned into several droplets and containing a high number of mitochondria [1]. Conversely, white adipocytes are large spherical cells that store energy-providing lipids (triglycerides) in the form of a unilocular droplet that occupies ~90% of the cell volume and can undergo lipolysis when required [1,14,21]. However, brown and white adipocytes coexist in the context of the adipose organ, and their relative amount depends upon several factors, such as age, gender, anatomical localization, and environmental stimuli such as temperature and nutritional status [21].

In recent years, several studies have reported that activation of the mineralocorticoid receptor (MR), a member of the nuclear receptor superfamily that mediates the response to mineralocorticoids (such as aldosterone) and, under appropriate conditions, to glucocorticoids [1], plays an essential role in the formation of WAT [22], while inhibition of MR promotes the conversion of white into brown adipocytes [23,24,25,26]. Moreover, MR activation induces the expression of pro-inflammatory genes such as interleukin 6 (IL6) and monocyte chemoattractant protein-1 (MCP-1), favoring macrophage type 1 infiltration and providing the environmental conditions for inflammation [25,27]. Conversely, MR inhibitors such as spironolactone reduce IL6 levels in patient serum [21,28].

In adipocytes, aldosterone, the MR ligand that elicits its transcriptional activation, has been reported to inhibit the expression and function of uncoupling protein-1 (UCP-1), thus favoring lipid storage at the expense of heat production [29]. On the contrary, MR antagonism induced UCP1 in WAT depots in vivo and markedly reduced the autophagic rate in murine preadipocytes in vitro, all events associated with adipose tissue browning [24,30]. Interestingly, dysregulation of autophagy contributes to aberrant differentiation of FPLD2 brown preadipocytes towards the white lineage [1,13]. In differentiating FPLD2 brown adipocyte precursors, we showed the formation of enlarged lipid droplets and the downregulation of brown adipose tissue genes [1]. Consistent with these observations, FPLD2 patient neck adipose tissue, although expressing the molecular markers of BAT [31], showed a white phenotype, and BAT activity was undetectable by PET-CT scan in an FPLD2 patient subjected to a cold test [1]. These data prompted us to consider the possibility that MR dysregulation could be involved in FPLD2 pathogenesis. Here, we identify lamin A as a new potential MR binding partner at the nuclear periphery that apparently affects MR distribution between the cytoplasm and the nucleus. Our results show aberrant nuclear import of MR in FPLD2 brown preadipocytes, a condition that contributes to their differentiation towards the white lineage, while treatment with the MR antagonist spironolactone redirects differentiation towards the brown lineage. These results and preliminary clinical observations in FPLD2 patients subjected to spironolactone suggest potential therapeutic treatments for *LMNA*-linked lipodystrophies.

## 2. Materials and Methods

*Cell cultures.* BAT samples were obtained from peri-thyroid biopsies of three healthy donors undergoing surgery for benign thyroid disorders or from FPLD2 patients carrying the R482Q-*LMNA* variant (two females, mean age 30 years) or the E202K-*LMNA* variant (female, age 30 years) undergoing aesthetic surgery in the peri-thyroid area [1]. Samples were used to establish brown adipocyte precursor cultures. The data were handled anonymously according to local and European ethical rules. All cell cultures belonged to the BioLaM biobank with the approval of the Rizzoli Orthopedic Institute Ethical Committee no. 0018250-2016. Pre-adipocytes were seeded in flasks and maintained in high-glucose Dulbecco’s modified Eagle’s medium (DMEM) (D5648, Sigma, St. Louis, MO, USA) supplemented with 20% fetal bovine serum (FBS) (10270-106, Thermo Fisher Scientific, Rodano (Milan), Italy), 100 IU/mL penicillin, and 100 µg/mL streptomycin (15140122, Thermo Fisher Scientific) (growth medium) in a 5% CO_2_ humidified atmosphere at 37 °C and allowed to reach confluence. From confluence, cells were transferred into DMEM containing 20% dextran-coated charcoal stripped fetal calf serum (DCC) (A3382101, Thermo Fisher Scientific).

*Brown adipocyte differentiation.* To achieve adipose differentiation, brown adipocyte precursors at day 2 post-confluence were shifted in induction medium composed of DMEM plus 10% DCC supplemented with 0.85 µM insulin (19278, Sigma), 0.2 nM triiodothyronine (T3) (T-2877, Sigma), 1 µM dexamethasone (DEXA) (D4902, Sigma), 0.5 mM isobutylmethylxanthine (IBMX) (I-5879, Sigma), and 125 nM indomethacin (I7378, Sigma). After 3 days of induction, the medium was replaced by brown differentiation medium (BAT medium): DMEM plus 10% DDC, 0.85 µM insulin, 20 nM T3, and 100 nM pioglitazone. Cells were maintained in BAT medium for 17 days, and the medium was replaced once a week.

*Compounds and treatments.* Spironolactone (S3378, Sigma) was used chronically at a concentration of 5 μM starting from day 3 of differentiation. N-Acetyl-S-farnesyl-L-cysteine-methyl ester (AFCMe; ALX-290-010-M010, Enzo Life Science, Armingdale, NY, USA) and mevinolin (M2147-25MG, Sigma) were used at concentrations of 50 μM and 25 μM, respectively, and treatments lasted 18 h. AFCMe is a non-competitor peptide that blocks the site of ZMPSTE24 endoprotease interaction in prelamin A, thus causing the accumulation of uncleaved farnesylated prelamin A [32]. Mevinolin is a statin and inhibits farnesyl production, thus reducing the available substrate for prelamin A farnesylation, in turn leading to the accumulation of non-farnesylated full-length prelamin A [32].

*Cell transfection.* HEK293 cells were transfected with MR-GFP alone or in combination with FLAG-tagged plasmids containing wild-type prelamin A (LA-WT), which undergoes normal maturation, and prelamin A carrying the pathogenetic variant R482Q causing FPLD2 (LA-R482Q), unprocessable prelamin A (LA-C661M, non-farnesylated prelamin A), or uncleavable prelamin A (LA-L647R, farnesylated prelamin A) [33]. Transfections were performed using Fugene transfection reagent (Invitrogen, Thermo Fisher Scientific) according to the manufacturer’s instructions. After transfection, cells were cultured for 24 h before fixation.

*Immunofluorescence analysis.* Cells grown on glass coverslips were fixed with 4% paraformaldheyde in PBS at 4 °C for 10 min and permeabilized using 70% methanol at room temperature for 3 min. After saturation of non-specific binding with PBS containing 4% BSA, coverslips were incubated with primary antibodies overnight at 4 °C and revealed with FITC- or TRIC-conjugated secondary antibodies (1 h at room temperature). Samples were mounted with an anti-fading reagent (Molecular Probes, Life Technologies, Monza, Italy) and observed with a Nikon ECLIPSE Ni epifluorescence microscope. Images captured by NIS-Elements 4.3 software were elaborated using Photoshop CS. NIS-Elements 4.3 software was also used for quantitative analysis of fluorescent signals.

*Western blot analysis*. After a short treatment with 10^−4^ M spirolonolactone or vehicle, cells were lysed in buffer containing 20 mM Tris-HCl, pH 7.5, 1% SDS, 1 mM Na_3_VO_4_, 1 mM PMSF, 5% 2-mercaptoethanol, and a phosphatase/protease inhibitor mix (Merck Millipore, Darmstadt, Germany), sonicated, and centrifuged for 30 min at 4 °C. Clarified lysates were diluted in Laemli buffer, subjected to SDS-PAGE (6%, 8%, or 4–20% gradient gel), and transferred to nitrocellulose membranes (3718, Santa Cruz, Dallas, TX, USA) overnight at 4 °C. Membranes were saturated with 4% BSA and incubated with primary antibodies for 1 h or overnight. Secondary antibodies were used at a 1:15,000 dilution for 20 min. Immunoblotted bands were revealed by ECL detection system (Thermo Fisher Scientific). Intensity measurement was performed using a Bio-Rad MP Imaging System, equipped with Image Lab Touch Software version 3.0.1.14 (Bio-Rad Laboratories Inc., Segrate, Milan, Italy).

*Nuclear fractioning.* To obtain nuclear fractions, cells detached with trypsin were incubated in a cold hypotonic buffer containing 10 mM Tris-HCl and protease inhibitors. Mechanical separation was then performed with a syringe, and centrifugation of nuclei was performed at 1100 r.p.m. for 10 min. Nuclei were lysed in buffer containing 50 mM Tris-HCl and 1% SDS containing or not 5% 2-mercaptoethanol. Lysates were subjected to SDS-PAGE and western blot analysis, as described above.

*Leptin and adiponectin analysis*. Cell culture supernatants were collected from adipocytes at 21 days in differentiation medium (72 h after medium replacement). The analysis of leptin and adiponectin release in the adipocyte supernatants was performed through multiplex bead-based sandwich immunoassay kits (Bio-Rad Laboratories Inc.) following the manufacturer instructions. Samples were analyzed by the Luminex Bio-plex system (Bio-Rad Laboratories Inc.). Data are reported as pg/mL.

*Proximity ligation assay.* In situ proximity ligation assay (PLA) was performed as previously described by using Duolink^®^In Situ Detection Reagent Orange (DUO92007, Sigma) [34]. Anti-lamin A polyclonal antibodies and anti-MR monoclonal antibodies were applied to detect lamin A-MR interactions in cells. Briefly, 4% paraformaldheyde-fixed samples were post-fixed in 70% methanol, treated with 4% BSA in PBS to block non-specific binding, and incubated with primary antibodies overnight at 4 °C. Thereafter, slides were incubated for 1 h at 37 °C with Duolink secondary probes (diluted to final concentrations of 1:5). Ligation solution was added to each sample, and slides were incubated in a humidity chamber for 30 min at 37 °C. Later, ligation solution was removed with wash buffer A, and amplification solution was added to each sample. Slides were incubated in a humidity chamber for 100 min at 37 °C and then washed with wash buffers. Duolink in situ mounting medium with DAPI was added to the slides, and samples were observed by a Nikon Eclipse Ni fluorescence microscope equipped with a digital CCD camera and NIS Elements AR 4.3 software. Quantitative analysis of PLA results was performed using Duolink Image Tool version 1.0.1.2 software (Sigma) by counting 100 nuclei per sample.

*Antibodies.* Antibodies employed for western blot analysis or immunofluorescence labeling were: anti-MR, mouse monoclonal (SC-53000, Santa Cruz Biotechnology); anti-lamin A rabbit polyclonal antibody (Ab26300, Abcam, Cambridge, UK); anti-prelamin A, goat polyclonal (SC-6214, Santa Cruz Biotechnology); anti-GAPDH, mouse monoclonal (MAB374, Merck Millipore); and anti-perilipin mouse monoclonal antibody (SC-390169, Santa Cruz Biotechnology).

*Statistical analysis.* Cell cultures from three FPLD2 patients carrying the above-reported variants were used in this study, and each experiment was performed on three biological replicates. The analysis of MFI was performed as follows: we selected at least 3 ROIs in the cytoplasm of pre-adipocytes or adipocytes and calculated the MFI per cell. We also selected the whole nucleus as the ROI. Where indicated in the legends, we calculated the ratio between nuclear and cytoplasmic MFIs. Experimental data are presented as means ± standard deviation (Std Dev). Statistical analyses were performed using the Prism 9 software (GraphPad Software, Version 10.0.3). All data analyzed, except those on cytokine levels, followed a Gaussian distribution and differences between groups were examined for statistical significance using parametric tests: a two-tailed Student’s *t*-test (for two groups) or a one-way Anova test (for multiple comparisons). A non-parametric Wilcoxon-Mann–Whitney test was used to analyze data on cytokine levels. Differences were considered statistically significant at *p* < 0.05.

*In situ modeling.* The primary amino acid sequences for the mineralocorticoid receptor (MCR_HUMAN) and prelamin A (*LMNA*_HUMAN) were retrieved from the UniProt/SwissProt database (accession numbers: P08235 and P02545, respectively). Protein structural modeling was conducted using AlfaFold Colab in multimer mode with a setting of 20 replicate cycles to generate the top five MCR:*LMNA* interactive models. AlfaFold generated predictions (.pdb files) were examined in the open-source program iCn3d available from the NCBI-NIH to visualize and determine sites of MCR:*LMNA* interaction. The open-source program Prodigy, available from Utrecht University (https://wenmr.science.uu.nl/prodigy/ accessed on 30 April 2023), was used to predict binding affinities and dissociation constants for each AlfaFold generated mode.

*In vivo PET study.* One female FPLD2 patient subjected to 6 months of treatment with spironolactone was enrolled in the in vivo PET/CT study with cold stimulation, and the results were compared to those obtained before the spironolactone treatment. The cold test protocol, PET-CT scanning, and image processing were conducted as previously described [1]. Briefly, the fasted patient was exposed to 2 h cold preconditioning, and then PET images were acquired dynamically with a PET/CT scanner (Discovery VCT, PET/CT, GE Healthcare, San Francisco, CA, USA). The glucose analog 18F-2-fluoro-2-deoxy-D-glucose (18F-FDG) was administered intravenously, and a dynamic emission scan was started simultaneously. After image correction and reconstruction, regions of interest (ROIs) were manually drawn in the fusion image composed of PET and CT images in all three transaxial, sagittal, and coronal planes to identify fat depots in the cheek, neck, and supraclavicular regions, on the trapezius muscle, and in the clavicular region corresponding to the subclavian vessel, representing the amount of tracer available in the circulation for organ extraction. The tissue extraction rate constant (Ki, mL/min/mL) was quantified by using the Patlak model [2]. Tissue glucose uptake (μmol/100 mL/min) was obtained by multiplying the Ki value by the mean plasma glucose concentration during PET and expressed per 100 mL of tissue volume.

## 3. Results

### 3.1. MR Localization

#### 3.1.1. MR Localization in FPLD2 Brown Preadipocytes

**Figure 1:**
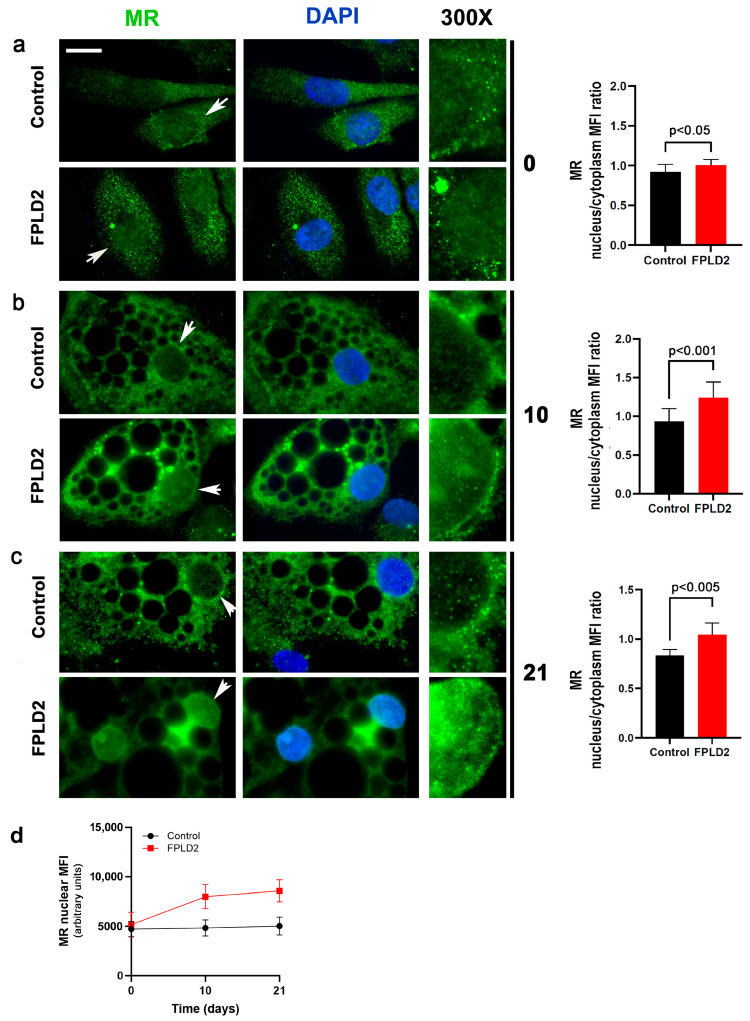
MR recruitment in FPLD2 brown adipocyte nuclei. MR staining by anti-MR antibody (green) is shown in control and FPLD2 cycling preadipocytes (**a**), differentiating preadipocytes (**b**), and adipocytes (**c**). Day of differentiation (0, 10 or 21) is indicated between images and the corresponding graph. 300× magnification of areas indicated by arrows is shown (300×). Nuclei are counterstained with DAPI. Bar, 10 µm. MR fluorescence intensity is reported in the graphs as (**a**–**c**) nuclear to cytoplasmic mean fluorescence intensity (MFI) ratio or (**d**) nuclear MFI. 50 cells per sample were analyzed in triplicate experiments. Statistically significant differences are reported in the graphs.

Adipocytes were derived from the neck adipose tissue of controls and FPLD2 patients. In the case of FPLD2, this adipose tissue can be considered an atypical BAT as it is mostly formed by white-like/beige adipocytes [1,3]. Both R482Q and E202K *LMNA* mutant cells were included in this study. As we obtained identical results with the three FPLD2 cell cultures except in the secretome analysis, representative images of MR localization, levels, and molecular interactions obtained with both cell cultures are shown throughout the manuscript. MR was localized in the cytoplasm and, to a lesser extent, in the nuclei of cycling control and FPLD2 brown preadipocytes (Figure 1a). However, at days 10 and 20 of differentiation, MR mean fluorescence intensity (MFI) was significantly increased in FPLD2 adipocyte nuclei but not in controls (Figure 1b–d, Figures S1 and S2).

These observations suggest that mutated prelamin A affects MR dynamics in FPLD2 cells.

#### 3.1.2. MR Localization in Cells Overexpressing Prelamin A

**Figure 2:**
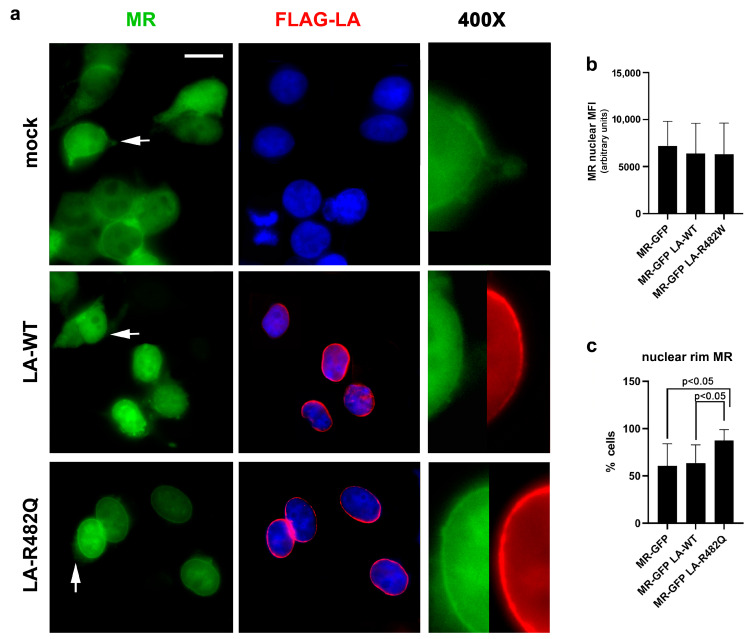
GFP-MR localization in cells overexpressing wild-type or mutated prelamin A. (**a**) GFP-MR localization (green) in HEK293 cells expressing GFP-MR in combination with a mock plasmid (mock), FLAG-LA-WT (LA-WT), or FLAG-LA-R482Q (LA-R482Q). Lamin A was labeled with an anti-FLAG antibody (red). Nuclei were counterstained with DAPI. 400× magnification of areas indicated by arrows is shown (400×). Bar, 10 µm. (**b**) Graphs represent the MFI of nuclear MR and (**c**) the percentage of nuclei showing accumulation of MR at the nuclear rim. 200 nuclei per sample were counted in triplicate experiments. Statistically significant differences are reported in the graphs.

To start addressing the potential role of prelamin A in MR nuclear recruitment, we expressed GFP-MR and FLAG-prelamin A in HEK293 cells. In the absence of any prelamin A plasmid, a variable degree of GFP-MR fluorescence signal was detected in nuclei, while the nuclear receptor was also localized in the cytoplasm (Figure 2a). Upon co-expression of wild-type lamin A (LA-WT), we did not observe a significant increase in the amount of nuclear MR, although cytoplasmic MR was reduced (Figure 2a,b). Moreover, the percentage of nuclei showing MR at the periphery was not different from mock-transfected cells (Figure 2c). As arginine 482 in the lamin A sequence is mutated in the majority of FPLD2 cases [3,9], we analyzed the effect of this *LMNA* variant (LA-R482Q) on MR localization (Figure 2a). When LA-R482Q was co-expressed with GFP-MR, the whole nuclear GFP signal was slightly but not significantly enhanced (Figure 2a,b), while a significantly increased percentage of nuclei showed GFP-MR accumulation in the nuclear periphery (Figure 2a,c). Of note, GFP-MR was not detectable in the cytoplasm of cells co-expressing LA-R482Q *LMNA* (Figure 2a).

**Figure 3:**
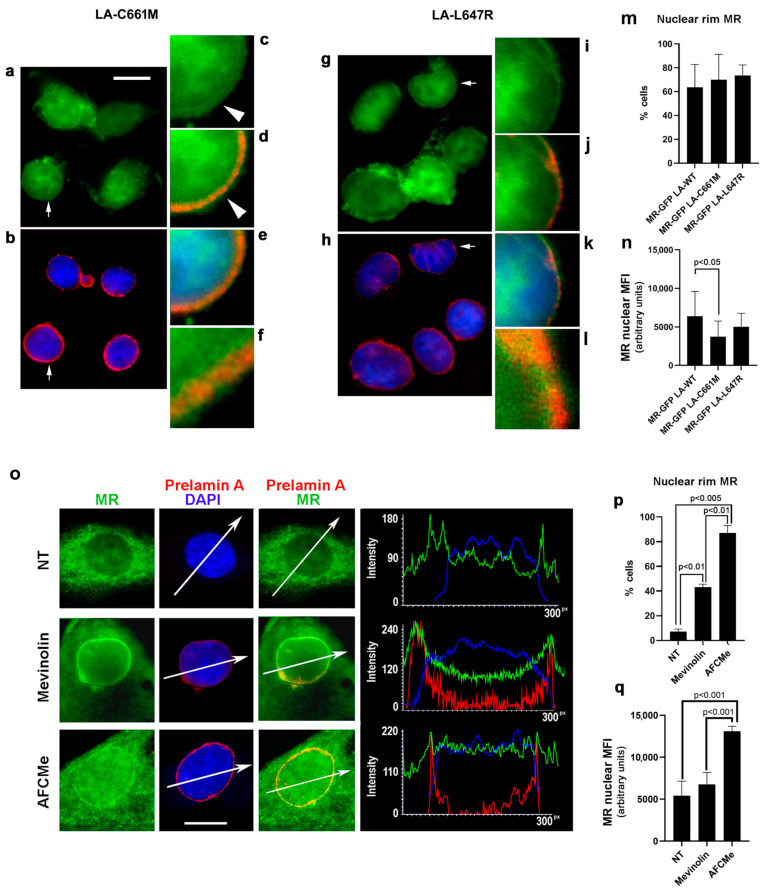
MR localization in nuclei that accumulate prelamin A. (**a**–**n**) GFP-MR localization (green) in HEK293 cells expressing GFP-MR in combination with FLAG-LA-C661M (LA-C661M) (**a**–**f**) or FLAG-LA-L647R (LA-L647R) (**g**–**n**). Prelamin A was revealed by anti-FLAG antibody (red). Nuclei were counterstained with DAPI. Arrows in (**a**,**b**) and (**g**,**h**) indicate areas selected for magnification (400×) in (**c**–**f**) and (**i**–**l**), respectively. Arrowheads indicate a double ring formed by MR at the nuclear periphery of cells expressing LA-C661M, which appears localized within the MR double ring (observed in (42.7/− ±6.5)% of transfected nuclei). (**m**,**n**) Graphs represent (**m**) the percentage of nuclei showing accumulation of MR at the nuclear rim and (**n**) MR nuclear MFI in transfected HEK293 cells. 200 nuclei per sample were counted in triplicate experiments. (**o**) Immunofluorescence staining of endogenous MR in normal human pre-adipocytes labeled with anti-MR monoclonal antibody (green) and anti-prelamin A polyclonal antibody (red). Cells were left untreated (NT), treated with mevinolin to accumulate non-farnesylated prelamin A (Mevinolin), or treated with AFCMe to accumulate farnesylated prelamin A (AFCMe). Bars, 10 µm. Representative mean fluorescence intensity profiles of each sample are shown on the right of each row. (**p**,**q**) Graphs represent (**p**) the percentage of nuclei showing accumulation of MR at the nuclear rim and (**q**) MR nuclear MFI in cells represented in (**o**). 50 nuclei per sample were counted in triplicate experiments. Statistically significant differences are indicated in the graphs.

As prelamin A tends to accumulate in FPLD2 cells [10,11,31,35], we analyzed MR fate in the presence of LA-C661M encoding the full-length unprocessable prelamin A, which is accumulated in the nuclear lamina as non-farnesylated prelamin A, or LA-L647R, which encodes an uncleavable permanently farnesylated prelamin A form [33]. Both prelamin A forms are accumulated in FPLD2 and other *LMNA*-linked lipodystrophies [36,37,38,39]. Overexpression of prelamin A increased the percentage of cells showing nuclear rim GFP-MR fluorescence relative to LA-WT-expressing cells, although the data did not reach statistical significance (Figure 3a–m vs. Figure 2a). In about 40% of LA-C661M-expressing nuclei, GFP-MR appeared as a double ring running along prelamin A (Figure 3c–f). However, overall nuclear GFP-MR MFI was decreased in LA-C661M cells but not in LA-L647R cells (Figure 3n). The degree of wild-type or mutant prelamin A colocalization with GFP-MR was determined using Pearson’s coefficient. The lowest colocalization coefficient was obtained in LA-C661M nuclei, while a coefficient of about 0.80 was determined for wild-type or mutant lamin A (Table S1).

The trend observed in transfected cells was supported by the analysis of mevinolin- and AFCMe-treated control human preadipocytes that accumulate non-farnesylated or farnesylated prelamin A, respectively. In fact, the percentage of cells showing MR at the nuclear envelope was significantly increased in cells accumulating both prelamin A forms (Figure 3o,p). Of note, in nuclei accumulating farnesylated prelamin A, MR MFI was overall increased (Figure 3q).

### 3.2. MR Binding to Prelamin A

**Figure 4:**
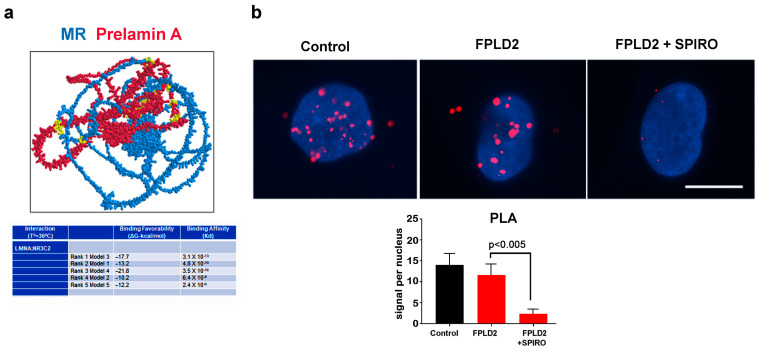
MR binding to lamin A in silico and in vivo. (**a**) In silico predictive analysis of the MR-prelamin A interaction using AlfaFold Colab, iCn3D (image; blue–MR; red–Prelamin A; yellow–points of protein:protein interaction) and Prodigy (table) web–based servers. (**b**) In situ proximity ligation assay (PLA) of MR and lamin A in control (control) and FPLD2 adipocytes either left untreated (FPLD2) or subjected to spironolactone (FPLD2 + SPIRO). Red dots (PLA signals) correspond to binding sites. The statistical analysis of PLA signals is reported in the graph and was performed by counting 100 nuclei per sample in triplicate experiments. Statistically significant differences are indicated in the graph.

As a whole, these results suggest a molecular interaction between MR and lamin A. In order to analyze the probability of a direct MR:lamin A interaction, we used AlfaFold Colab to predict the structure of the MR:lamin A interaction and then analyzed the generated predicted models in iCn3d (https://www.ncbi.nlm.nih.gov/Struture/icn3d/full.html accessed on 2 May 2023) and Prodigy (https://wenmr.science.uu.nl/prodigy/ accessed on 30 April 2023) to determine the sites of interaction and binding affinities of the predicted interaction. Most predicted MR:lamin A models indicated the potential for a strong association between these two proteins (Figure 4a). In vivo binding of MR to lamin A was suggested by the in situ proximity ligation assay (PLA) (Figure 4b). Lamin A-MR PLA signals were detected both in control and FPLD2 adipocytes, and the number of signals was not significantly different in FPLD2 (Figure 4b). Nevertheless, inhibition of MR dimerization by spironolactone almost completely abolished the lamin A-MR interaction (Figure 4b).

### 3.3. MR Antagonism Redirects FPLD2 Brown Preadipocyte Differentiation

**Figure 5:**
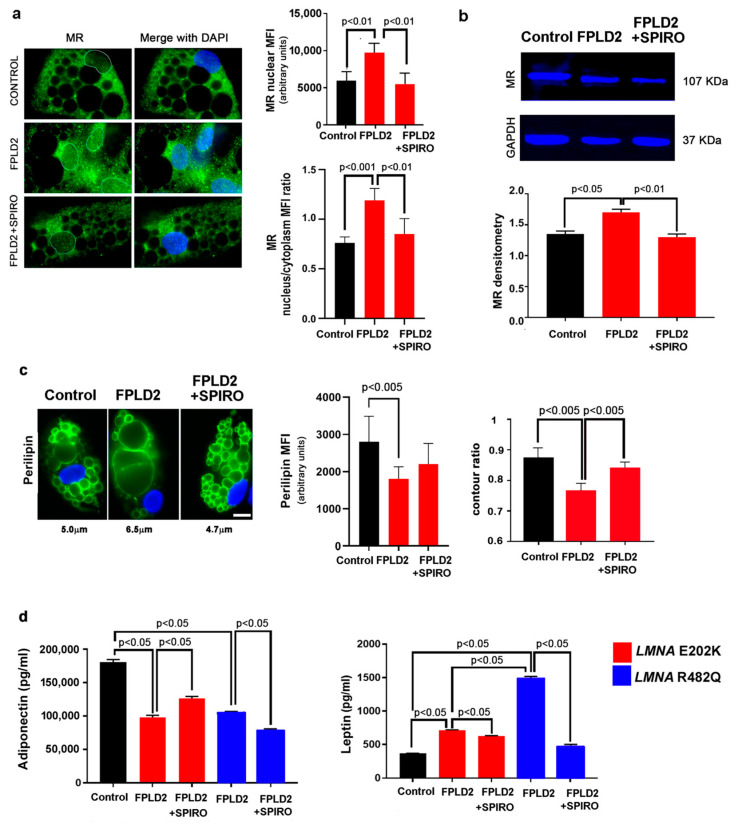
Spironolactone redirects the differentiation of FPLD2 brown adipocyte precursors towards the brown lineage. (**a**) Representative images of MR immunofluorescence localization in control (Control) and FPLD2 cells at day 21 during BAT differentiation. FPLD2 cells were left untreated (FPLD2) or treated with spironolactone (FPLD2 + SPIRO). Nuclear MR MFI and the nuclear:cytoplasmic MFI ratio are reported in the graphs. (**b**) MR protein amount in adipocyte nuclei was measured by western blot analysis. MR densitometry is reported in the graph after normalization to GAPDH as a loading control. (**c**) Adipocytes stained for perilipin using anti-perilipin antibody (green) and DAPI (blue). Mean vesicle diameter is indicated below the corresponding images. Bars, 10 μm. Perilipin levels (Perilipin MFI) and lipid vesicle dysmorphism (contour ratio) are shown in the graphs. (**d**) Leptin and adiponectin levels in control (Control) and FPLD2 brown adipocytes left untreated (FPLD2) or treated with spironolactone (FPLD2 + SPIRO). Leptin and adiponectin levels in the adipocyte secretome are reported in the graphs. Experiments were done in triplicate. Fifty cells per sample were analyzed in (**a**,**c**). Statistically significant differences are indicated in the graphs.

However, consistent with an increased nuclear:cytoplasmic MR ratio, FPLD2 brown pre-adipocytes tended to differentiate towards the white lineage, as previously reported [1,14] and here confirmed by the analysis of mitochondrial uncoupling activity showing reduced proton leak in FPLD2 adipocytes (Figure S3). To test if this condition could be related to MR activation, we inhibited MR by using its antagonist, spironolactone, which impairs MR dimerization and nuclear shuttling [24]. In FPLD2 brown adipocytes, long-term spironolactone treatment reduced the MR nuclear amount and avoided the formation of enlarged lipid droplets (Figure 5a,b). Moreover, FPLD2 brown adipocytes formed dysmorphic lipid droplets with reduced perilipin levels relative to controls (Figure 5c). High perilipin levels are associated with brown adipogenesis [40,41]. Since perilipin tended to increase upon spironolactone treatment (Figure 5c), these observations supported a MR-related whitening of FPLD2 brown preadipocytes.

Of note, spironolactone also avoided lipid droplet dysmorphism (Figure 5c). MR activation is known to affect the cellular secretome by reducing adiponectin release and increasing leptin levels in adipocytes [42]. Accordingly, in FPLD2 brown adipocytes, adiponectin secretion was reduced and leptin secretion increased relative to controls (Figure 5d). However, we observed a different behavior in FPLD2 cells carrying different *LMNA* mutations. In fact, while adiponectin was decreased in the secretome of all cell cultures, spironolactone elicited an increase in adiponectin in *LMNA*-*E202K* adipocytes and a decrease in *LMNA*-*R482Q* cells (Figure 5d). On the other hand, while leptin levels were increased in all FPLD2 samples, much higher levels were measured in *LMNA*-*R482Q* cells (Figure 5d). Spironolactone significantly inhibited leptin secretion in all FPLD2 cell cultures (Figure 5d). Spironolactone effects on leptin and adiponectin secretion further supported MR involvement in the aberrant differentiation of FPLD2 brown preadipocytes [43].

### 3.4. MR Antagonism in FPLD2

Based on the experimental evidence reported above, we started to evaluate the effects of MR antagonism in FPLD2 patients undergoing spironolactone therapy to counteract hyperandrogenism and other metabolic abnormalities. We had previously reported that adipose depots located in supraclavicular regions, the face, and the neck of FPLD2 patients do not show cold-induced activation as measured by PET-CT with ^18^F-FDG during a cold test [1]; such activation is a typical feature of functional BAT in healthy adults [20].

**Figure 6:**
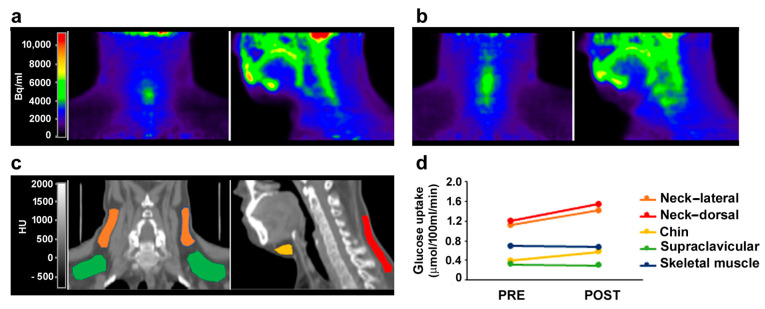
Glucose uptake in a FPLD2 patient undergoing spironolactone treatment. PET-CT images of one FPLD2 patient before (**a**) and after 6-months of spironolactone treatment (**b**) are shown. The patient’s neck and head (up to the nose) are visualized in coronal (**left panels**) and sagittal (**right panels**) planes in PET (**a**,**b**) and co-registered CT (**c**) images for anatomical reference. PET images show tissue radioactivity, expressed as becquerel per ml of tissue (Bq/mL) (**a**), whereas CT images show tissue radiodensity expressed in Hounsfield units (HU). Several regions of interest (ROIs) corresponding to fat depots of the neck (lateral and dorsal), chin, and supraclavicular regions were identified in co-registered CT images (**c**), and ^18^F-FDG extraction rates were measured in PET images. Glucose uptake was calculated as the product of ^18^F-FDG extraction rate and glycemia during the PET scan. Pre- vs. post-treatment levels of cold-induced glucose uptake in these fat depots were compared (**d**). Glucose uptake in skeletal muscle (trapezius muscle) was also analyzed for comparison as a negative control. The colors in the CT image and in the graph correspond to the anatomical identification of the adipose depots analyzed.

In a female FPLD2 patient subjected to 6 months of treatment with spironolactone at a dose of 100 mg per day for hyperandrogenism, the ^18^F-FDG PET-CT imaging analysis during the cold test was repeated, and the results were compared to those calculated before the spironolactone treatment. In the post-compared to the pre-treatment condition, glucose uptake during cold stimulation was increased in adipose depots in the neck regions, lateral and dorsal depots, and in the chin, but not in skeletal muscle, in which glucose uptake is not affected in the cold test (Figure 6). These data indicate that spironolactone treatment increased cold-stimulated activity in the above-mentioned adipose depots, which is suggestive of browning.

## 4. Discussion

Our study shows the involvement of lamin A in MR recruitment to the nucleus and adds altered MR dynamics to the complex scenario of FPLD2 pathogenetic pathways. Our in silico study predicts a strong affinity between prelamin A and MR, and the in situ PLA suggests that MR directly binds prelamin A in control and FPLD2 adipocytes, although the binding seems to be abolished by spironolactone. The latter observation raises the possibility that only the active form of MR, which is in most cases a homodimer, is able to bind lamin A. Our results do not show an altered binding of mutated prelamin A to MR, suggesting that the increased nuclear import observed in FPLD2 brown adipocytes could involve other, possibly tissue-specific interactors [44]. Of note, a major player in the MR docking platform is the heat-shock protein 90 (HSP90) [44], a lamin A-binding molecule [45,46] affected in progeroid laminopathies featuring lipodystrophy [47]. Moreover, MR nuclear import requires dynein [48], a motor protein that associates with the linker of the nucleoskeleton and cytoskeleton (LINC) complex, a main lamin A-interacting platform [49,50]. On the other hand, prelamin A interaction with ubiquitous [50] or adipocyte-specific nuclear envelope proteins, such as TMEM120A [14], could play a role in MR recruitment to the nucleus under normal or pathological conditions. Alternatively, corticosteroid rapid signaling in the cytoplasm could be involved in lamin A-dependent MR nuclear shuttling [51], a possibility that needs further investigation. In differentiating preadipocytes and mature brown adipocytes, the increased MR nuclear import establishes a condition resembling white adipose tissue cells. Accordingly, as previously reported [1,14], FPLD2 brown adipocytes form enlarged lipid droplets. Moreover, increased leptin secretion is observed, supporting a white adipocyte feature. As this condition is counteracted by the MR antagonist spironolactone, which also impairs MR-lamin A interaction, proper lamin A-MR interplay appears to play a relevant role in the maintenance of the brown phenotype.

Despite some differences in the recovery of adipokine levels, the efficacy of spironolactone in redirecting differentiation of FPLD2 brown adipocytes carrying different *LMNA* mutations suggests that MR inhibition could counteract the accumulation of ectopic white adipose tissue in FPLD2 patients’ necks. In fact, here we show that spironolactone treatment avoids aberrant MR nuclear import in brown FPLD2 adipocytes, their aberrant whitening in culture, and appears to improve BAT cold-induced activity in vivo. These findings suggest spironolactone as a potential therapeutic tool for FPLD2 and possibly other lipodystrophic laminopathies. This possibility needs to be tested by evaluating MR fate and dynamics in white FPLD2 adipocytes and in vivo models of *LMNA*-linked lipodystrophy and assaying the eventual effect of spironolactone in those experimental models. Our study also shows that drugs inducing non-farnesylated and mostly farnesylated prelamin A trigger MR recruitment to the nuclear envelope, suggesting that prelamin A has a higher affinity for MR relative to mature lamin A. However, given the stronger effect of farnesylated prelamin A, we can speculate that inhibition of prelamin A farnesylation by statins or lonafarnib [33] might restore a more physiological condition in FPLD2 brown adipocytes and other *LMNA*-linked lipodystrophies featuring farnesylated prelamin A accumulation, such as Mandibuloacral Dysplasia and Hutchinson-Gilford Progeria [52]. Of note, MR is a trigger of IL6 expression, a main determinant of *LMNA*-linked lipodystrophy [17,53]. In our hands, inconsistent data on MR-dependent IL6 expression and secretion were obtained in different FPLD2 patients, which have not been reported in this paper. We are deepening this key relationship in adipocytes and other cell types from different laminopathic preclinical models. In that context, the fundamental regulatory role that MR plays in different tissues and organs must also be considered. As endothelial cells, vascular smooth muscle cells, and cardiomyocytes are the main targets of MR activity, the investigation of MR fate in laminopathies—all featuring cardiovascular and/or cardiac disorders—appears of utmost importance.

## Data Availability

Data supporting reported results can be provided by the authors upon reasonable request.

## Supplementary Materials

The following supporting information can be downloaded at: https://www.mdpi.com/article/10.3390/cells12222586/s1, Table S1: Analysis of MR-lamin A co-localization. Figure S1: MR staining in control adipocytes. Figure S2: MR staining in FPLD2 adipocytes. Figure S3: Bioenergetic metabolism in control and FPLD2 brown adipocytes.

## References

[1] Pellegrini C., Columbaro M., Schena E., Prencipe S., Andrenacci D., Iozzo P., Angela Guzzardi M., Capanni C., Mattioli E., Loi M. (2019). Altered adipocyte differentiation and unbalanced autophagy in type 2 Familial Partial Lipodystrophy: An in vitro and in vivo study of adipose tissue browning. Exp. Mol. Med..

[2] Shackleton S., Lloyd D.J., Jackson S.N., Evans R., Niermeijer M.F., Singh B.M., Schmidt H., Brabant G., Kumar S., Durrington P.N. (2000). LMNA, encoding lamin A/C, is mutated in partial lipodystrophy. Nat. Genet..

[3] Fernandez-Pombo A., Diaz-Lopez E.J., Castro A.I., Sanchez-Iglesias S., Cobelo-Gomez S., Prado-Morana T., Araujo-Vilar D. (2023). Clinical Spectrum of LMNA-Associated Type 2 Familial Partial Lipodystrophy: A Systematic Review. Cells.

[4] von Schnurbein J., Adams C., Akinci B., Ceccarini G., D’Apice M.R., Gambineri A., Hennekam R.C.M., Jeru I., Lattanzi G., Miehle K. (2020). European lipodystrophy registry: Background and structure. Orphanet J. Rare Dis..

[5] Araujo-Vilar D., Fernandez-Pombo A., Victoria B., Mosquera-Orgueira A., Cobelo-Gomez S., Castro-Pais A., Hermida-Ameijeiras A., Loidi L., Sanchez-Iglesias S. (2021). Variable Expressivity and Allelic Heterogeneity in Type 2 Familial Partial Lipodystrophy: The p.(Thr528Met) LMNA Variant. J. Clin. Med..

[6] Araujo-Vilar D., Lado-Abeal J., Palos-Paz F., Lattanzi G., Bandin M.A., Bellido D., Dominguez-Gerpe L., Calvo C., Perez O., Ramazanova A. (2008). A novel phenotypic expression associated with a new mutation in LMNA gene, characterized by partial lipodystrophy, insulin resistance, aortic stenosis and hypertrophic cardiomyopathy. Clin. Endocrinol..

[7] Gambineri A., Semple R.K., Forlani G., Genghini S., Grassi I., Hyden C.S., Pagotto U., O’Rahilly S., Pasquali R. (2008). Monogenic polycystic ovary syndrome due to a mutation in the lamin A/C gene is sensitive to thiazolidinediones but not to metformin. Eur. J. Endocrinol..

[8] Mosbah H., Donadille B., Vatier C., Janmaat S., Atlan M., Badens C., Barat P., Beliard S., Beltrand J., Ben Yaou R. (2022). Dunnigan lipodystrophy syndrome: French National Diagnosis and Care Protocol (PNDS; Protocole National de Diagnostic et de Soins). Orphanet J. Rare Dis..

[9] Araujo-Vilar D., Santini F. (2019). Diagnosis and treatment of lipodystrophy: A step-by-step approach. J. Endocrinol. Investig..

[10] Araujo-Vilar D., Lattanzi G., Gonzalez-Mendez B., Costa-Freitas A.T., Prieto D., Columbaro M., Mattioli E., Victoria B., Martinez-Sanchez N., Ramazanova A. (2009). Site-dependent differences in both prelamin A and adipogenic genes in subcutaneous adipose tissue of patients with type 2 familial partial lipodystrophy. J. Med. Genet..

[11] Capanni C., Mattioli E., Columbaro M., Lucarelli E., Parnaik V.K., Novelli G., Wehnert M., Cenni V., Maraldi N.M., Squarzoni S. (2005). Altered pre-lamin A processing is a common mechanism leading to lipodystrophy. Hum. Mol. Genet..

[12] Afonso P., Auclair M., Boccara F., Vantyghem M.C., Katlama C., Capeau J., Vigouroux C., Caron-Debarle M. (2016). LMNA mutations resulting in lipodystrophy and HIV protease inhibitors trigger vascular smooth muscle cell senescence and calcification: Role of ZMPSTE24 downregulation. Atherosclerosis.

[13] Infante A., Gago A., de Eguino G.R., Calvo-Fernandez T., Gomez-Vallejo V., Llop J., Schlangen K., Fullaondo A., Aransay A.M., Martin A. (2014). Prelamin A accumulation and stress conditions induce impaired Oct-1 activity and autophagy in prematurely aged human mesenchymal stem cell. Aging.

[14] Czapiewski R., Batrakou D.G., de Las Heras J.I., Carter R.N., Sivakumar A., Sliwinska M., Dixon C.R., Webb S., Lattanzi G., Morton N.M. (2022). Genomic loci mispositioning in Tmem120a knockout mice yields latent lipodystrophy. Nat. Commun..

[15] Oldenburg A.R., Delbarre E., Thiede B., Vigouroux C., Collas P. (2014). Deregulation of Fragile X-related protein 1 by the lipodystrophic lamin A p.R482W mutation elicits a myogenic gene expression program in preadipocytes. Hum. Mol. Genet..

[16] Oldenburg A., Briand N., Sorensen A.L., Cahyani I., Shah A., Moskaug J.O., Collas P. (2017). A lipodystrophy-causing lamin A mutant alters conformation and epigenetic regulation of the anti-adipogenic MIR335 locus. J. Cell Biol..

[17] Hartinger R., Lederer E.M., Schena E., Lattanzi G., Djabali K. (2023). Impact of Combined Baricitinib and FTI Treatment on Adipogenesis in Hutchinson–Gilford Progeria Syndrome and Other Lipodystrophic Laminopathies. Cells.

[18] Kwok K.H., Lam K.S., Xu A. (2016). Heterogeneity of white adipose tissue: Molecular basis and clinical implications. Exp. Mol. Med..

[19] Giordano A., Smorlesi A., Frontini A., Barbatelli G., Cinti S. (2014). White, brown and pink adipocytes: The extraordinary plasticity of the adipose organ. Eur. J. Endocrinol..

[20] Cypess A.M., Lehman S., Williams G., Tal I., Rodman D., Goldfine A.B., Kuo F.C., Palmer E.L., Tseng Y.H., Doria A. (2009). Identification and importance of brown adipose tissue in adult humans. N. Engl. J. Med..

[21] Cinti S. (2018). Adipose Organ Development and Remodeling. Compr. Physiol..

[22] Gomez-Sanchez C.E. (2015). What Is the Role of the Adipocyte Mineralocorticoid Receptor in the Metabolic Syndrome?. Hypertension.

[23] Armani A., Marzolla V., Fabbri A., Caprio M. (2015). Cellular mechanisms of MR regulation of adipose tissue physiology and pathophysiology. J. Mol. Endocrinol..

[24] Armani A., Cinti F., Marzolla V., Morgan J., Cranston G.A., Antelmi A., Carpinelli G., Canese R., Pagotto U., Quarta C. (2014). Mineralocorticoid receptor antagonism induces browning of white adipose tissue through impairment of autophagy and prevents adipocyte dysfunction in high-fat-diet-fed mice. FASEB J..

[25] Hirata A., Maeda N., Hiuge A., Hibuse T., Fujita K., Okada T., Kihara S., Funahashi T., Shimomura I. (2009). Blockade of mineralocorticoid receptor reverses adipocyte dysfunction and insulin resistance in obese mice. Cardiovasc. Res..

[26] Feraco A., Marzolla V., Scuteri A., Armani A., Caprio M. (2020). Mineralocorticoid Receptors in Metabolic Syndrome: From Physiology to Disease. Trends Endocrinol. Metab..

[27] Infante M., Armani A., Mammi C., Fabbri A., Caprio M. (2017). Impact of Adrenal Steroids on Regulation of Adipose Tissue. Compr. Physiol..

[28] Corbould A. (2007). Effects of spironolactone on glucose transport and interleukin-6 secretion in adipose cells of women. Horm. Metab. Res..

[29] Marzolla V., Feraco A., Limana F., Kolkhof P., Armani A., Caprio M. (2022). Class-specific responses of brown adipose tissue to steroidal and nonsteroidal mineralocorticoid receptor antagonists. J. Endocrinol. Investig..

[30] Liu K., Czaja M.J. (2013). Regulation of lipid stores and metabolism by lipophagy. Cell Death Differ..

[31] Bereziat V., Cervera P., Le Dour C., Verpont M.C., Dumont S., Vantyghem M.C., Capeau J., Vigouroux C., Lipodystrophy Study G. (2011). LMNA mutations induce a non-inflammatory fibrosis and a brown fat-like dystrophy of enlarged cervical adipose tissue. Am. J. Pathol..

[32] Mattioli E., Columbaro M., Capanni C., Santi S., Maraldi N.M., D’Apice M.R., Novelli G., Riccio M., Squarzoni S., Foisner R. (2008). Drugs affecting prelamin A processing: Effects on heterochromatin organization. Exp. Cell Res..

[33] Capanni C., Schena E., Di Giampietro M.L., Montecucco A., Mattioli E., Lattanzi G. (2022). The role of prelamin A post-translational maturation in stress response and 53BP1 recruitment. Front. Cell Dev. Biol..

[34] Santi S., Cenni V., Capanni C., Lattanzi G., Mattioli E. (2020). PCAF Involvement in Lamin A/C-HDAC2 Interplay during the Early Phase of Muscle Differentiation. Cells.

[35] Caron M., Auclair M., Donadille B., Bereziat V., Guerci B., Laville M., Narbonne H., Bodemer C., Lascols O., Capeau J. (2007). Human lipodystrophies linked to mutations in A-type lamins and to HIV protease inhibitor therapy are both associated with prelamin A accumulation, oxidative stress and premature cellular senescence. Cell Death Differ..

[36] Dominici S., Fiori V., Magnani M., Schena E., Capanni C., Camozzi D., D’Apice M.R., Le Dour C., Auclair M., Caron M. (2009). Different prelamin A forms accumulate in human fibroblasts: A study in experimental models and progeria. Eur. J. Histochem..

[37] Le Dour C., Schneebeli S., Bakiri F., Darcel F., Jacquemont M.L., Maubert M.A., Auclair M., Jeziorowska D., Reznik Y., Bereziat V. (2011). A homozygous mutation of prelamin-A preventing its farnesylation and maturation leads to a severe lipodystrophic phenotype: New insights into the pathogenicity of nonfarnesylated prelamin-A. J. Clin. Endocrinol. Metab..

[38] Andre P., Schneebeli S., Vigouroux C., Lascols O., Schaaf M., Chevalier P. (2015). Metabolic and cardiac phenotype characterization in 37 atypical Dunnigan patients with nonfarnesylated mutated prelamin A. Am. Heart J..

[39] Camozzi D., D’Apice M.R., Schena E., Cenni V., Columbaro M., Capanni C., Maraldi N.M., Squarzoni S., Ortolani M., Novelli G. (2012). Altered chromatin organization and SUN2 localization in mandibuloacral dysplasia are rescued by drug treatment. Histochem. Cell Biol..

[40] Sawada T., Miyoshi H., Shimada K., Suzuki A., Okamatsu-Ogura Y., Perfield J.W., Kondo T., Nagai S., Shimizu C., Yoshioka N. (2010). Perilipin overexpression in white adipose tissue induces a brown fat-like phenotype. PLoS ONE.

[41] Souza S.C., Christoffolete M.A., Ribeiro M.O., Miyoshi H., Strissel K.J., Stancheva Z.S., Rogers N.H., D’Eon T.M., Perfield J.W., Imachi H. (2007). Perilipin regulates the thermogenic actions of norepinephrine in brown adipose tissue. J. Lipid Res..

[42] Guo C., Ricchiuti V., Lian B.Q., Yao T.M., Coutinho P., Romero J.R., Li J., Williams G.H., Adler G.K. (2008). Mineralocorticoid receptor blockade reverses obesity-related changes in expression of adiponectin, peroxisome proliferator-activated receptor-gamma, and proinflammatory adipokines. Circulation.

[43] Martinez-Sanchez N. (2020). There and Back Again: Leptin Actions in White Adipose Tissue. Int. J. Mol. Sci..

[44] Jimenez-Canino R., Lorenzo-Diaz F., Jaisser F., Farman N., Giraldez T., Alvarez de la Rosa D. (2016). Histone Deacetylase 6-Controlled Hsp90 Acetylation Significantly Alters Mineralocorticoid Receptor Subcellular Dynamics But Not its Transcriptional Activity. Endocrinology.

[45] Wang Y., Chen Q., Wu D., Chen Q., Gong G., He L., Wu X. (2021). Lamin-A interacting protein Hsp90 is required for DNA damage repair and chemoresistance of ovarian cancer cells. Cell Death Dis..

[46] Thanomkitti K., Fong-Ngern K., Sueksakit K., Thuangtong R., Thongboonkerd V. (2018). Molecular functional analyses revealed essential roles of HSP90 and lamin A/C in growth, migration, and self-aggregation of dermal papilla cells. Cell Death Discov..

[47] Hamczyk M.R., Villa-Bellosta R., Quesada V., Gonzalo P., Vidak S., Nevado R.M., Andres-Manzano M.J., Misteli T., Lopez-Otin C., Andres V. (2019). Progerin accelerates atherosclerosis by inducing endoplasmic reticulum stress in vascular smooth muscle cells. EMBO Mol. Med..

[48] Galigniana M.D., Erlejman A.G., Monte M., Gomez-Sanchez C., Piwien-Pilipuk G. (2010). The hsp90-FKBP52 complex links the mineralocorticoid receptor to motor proteins and persists bound to the receptor in early nuclear events. Mol. Cell. Biol..

[49] Vahabikashi A., Sivagurunathan S., Nicdao F.A.S., Han Y.L., Park C.Y., Kittisopikul M., Wong X., Tran J.R., Gundersen G.G., Reddy K.L. (2022). Nuclear lamin isoforms differentially contribute to LINC complex-dependent nucleocytoskeletal coupling and whole-cell mechanics. Proc. Natl. Acad. Sci. USA.

[50] Verstraeten V.L., Renes J., Ramaekers F.C., Kamps M., Kuijpers H.J., Verheyen F., Wabitsch M., Steijlen P.M., van Steensel M.A., Broers J.L. (2011). Reorganization of the nuclear lamina and cytoskeleton in adipogenesis. Histochem. Cell Biol..

[51] Ong G.S., Young M.J. (2017). Mineralocorticoid regulation of cell function: The role of rapid signalling and gene transcription pathways. J. Mol. Endocrinol..

[52] Cenni V., Capanni C., Mattioli E., Schena E., Squarzoni S., Bacalini M.G., Garagnani P., Salvioli S., Franceschi C., Lattanzi G. (2020). Lamin A involvement in ageing processes. Ageing Res. Rev..

[53] Squarzoni S., Schena E., Sabatelli P., Mattioli E., Capanni C., Cenni V., D’Apice M.R., Andrenacci D., Sarli G., Pellegrino V. (2021). Interleukin-6 neutralization ameliorates symptoms in prematurely aged mice. Aging Cell.

