# Supplementary material for: Mineralocorticoid Receptor Antagonism Prevents Type 2 Familial Partial Lipodystrophy Brown Adipocyte Dysfunction
Source: Cells. 2023 Nov 7;12(22):2586. doi: 10.3390/cells12222586 (PMC10670260; doi:10.3390/cells12222586)
Supplement: Supplementary file 1 [file cells-12-02586-s001.zip › cells-2628987-supplementary.pdf]

cells

MDPI

SUPPLEMENTARY MATERIAL

Table S1. Analysis of MR-lamin A colocalization determined as Pearson's Correlation Coefficient in HEK293 cells co-expressing GFP-MR and FLAG-lamin A. Thirty nuclei per sample were analyzed in triplicate experiments.

**Pearson's Coefficient**	**mean**	**SD**
LA-WT	0,80	0,09
LA-C661M	0,69	0,19
LA-R482Q	0,81	0,10
LA-R647R	0,86	0,07

cells

Figure S1

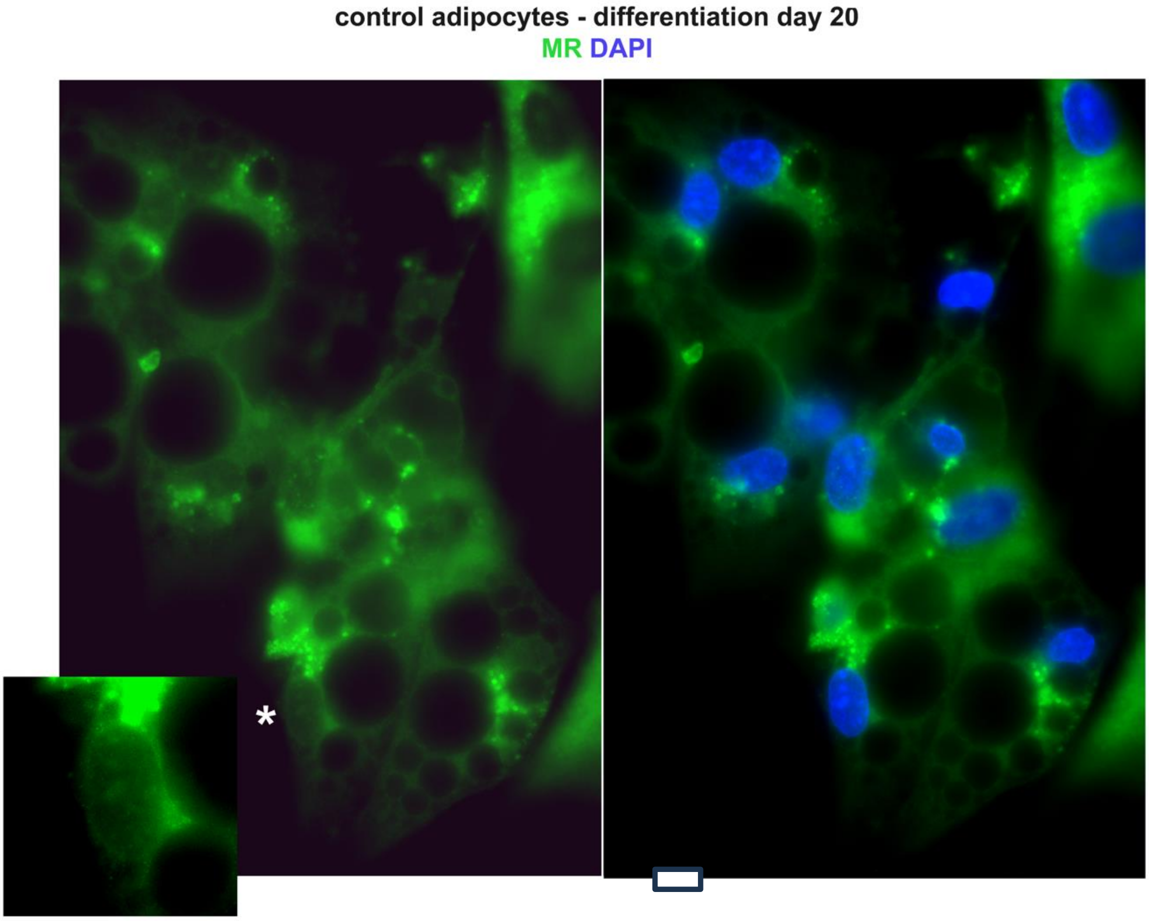

Figure S1. MR staining in control adipocytes. MR was labeled by an anti-MR monoclonal antibody. Nuclei are counterstained with DAPI. Lipid vesicles appear Bar, 10 mm. The white asterisk indicates the nucleus shown in the inset.

cells

MDPI

Figure S2

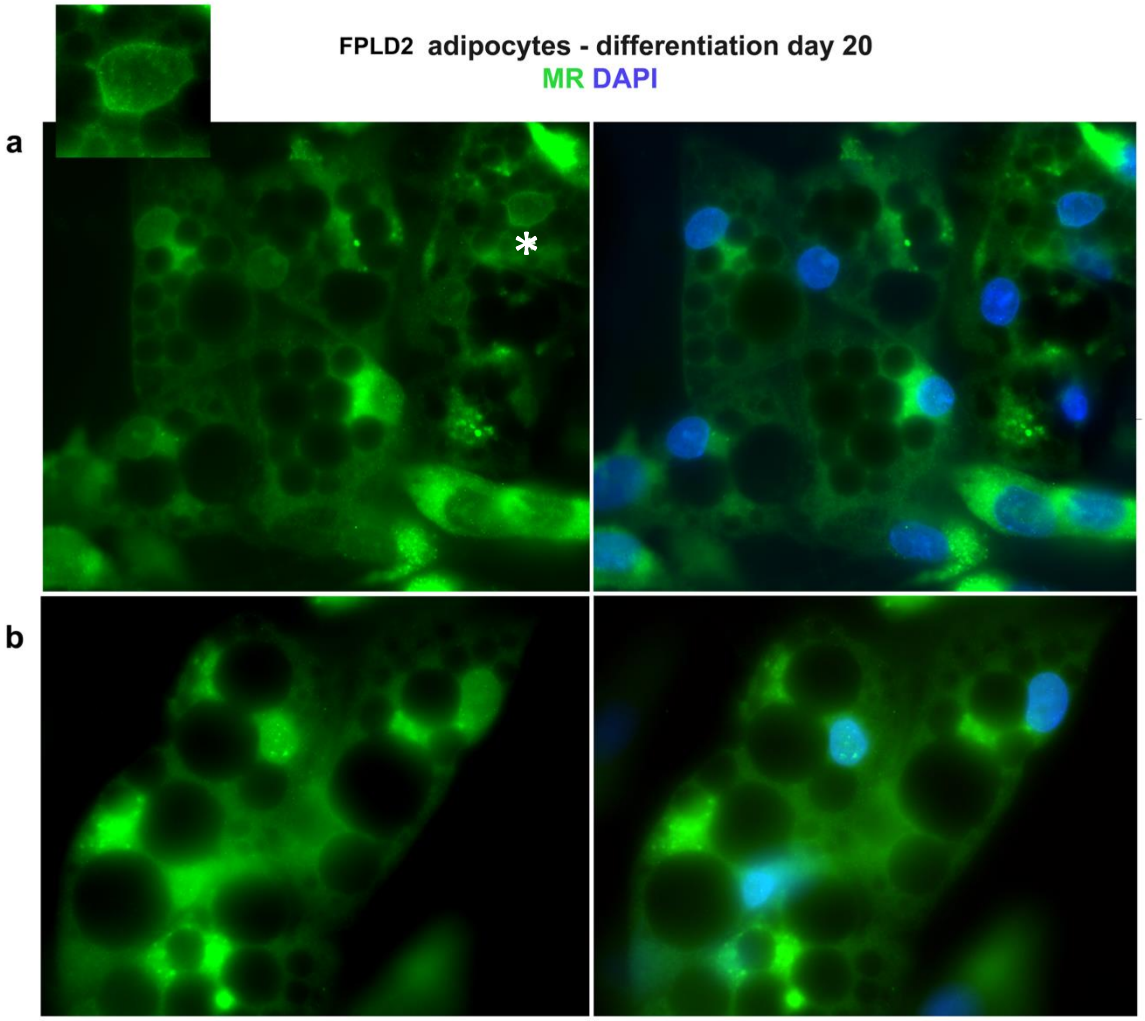

Figure S2. MR staining in FPLD2 adipocytes. (a) Adipocytes obtained from a patient carrying the R482Q-LMNA nutation. (b) Adipocytes obtained from a patient carrying the E202K-LMNA nutation, Bar, 10 mm. The white asterisk indicates the nucleus shown in the inset.

cells

MDPI

Figure S3

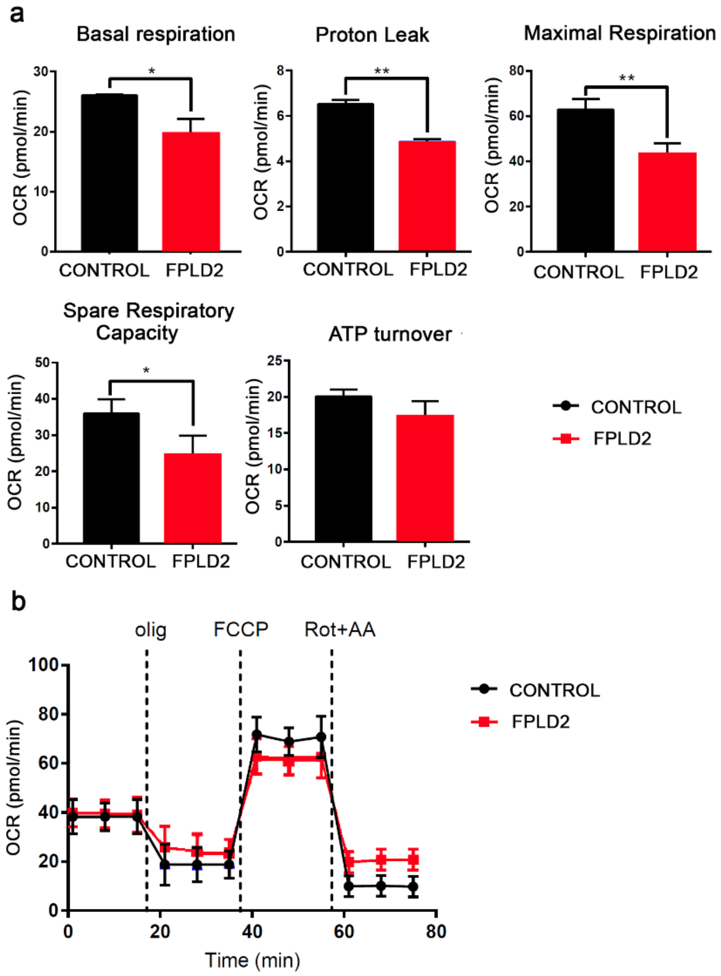

Figure S3. Bioenergetic metabolism in control and FPLD2 brown adipocytes. (a) Mitochondrial parameters (basal respiration,Proton leak, maximal respiration, spare respiratory capacity and ATP production) of control (CONTROL) and FPLD2 brown adipocytes are reported in the graphs. (b) The mitochondrial respiration profile in control (CONTROL) and FPLD2 brown adipocytes was evaluated as OCR (Oxygen Consumption Rate) under basal respiration conditions. Statistically significant differences are indicated (*p<0.05; **p<0.01).
